# Repeat disturbances have cumulative impacts on stream communities

**DOI:** 10.1002/ece3.4968

**Published:** 2019-02-14

**Authors:** Jessica M. Haghkerdar, Jack R. McLachlan, Alexis Ireland, Hamish S. Greig

**Affiliations:** ^1^ School of Biology and Ecology University of Maine Orono Maine; ^2^ Centre for Biological Diversity University of St Andrews St Andrews UK; ^3^ Ecology and Environmental Sciences Program University of Maine Orono Maine

**Keywords:** community composition, diversity, dominance, resilience, vulnerability

## Abstract

1. Climate change has altered disturbance regimes in many ecosystems, and predictions show that these trends are likely to continue. The frequency of disturbance events plays a particularly important role in communities by selecting for disturbance‐tolerant taxa.
2. However, ecologists have yet to disentangle the influence of disturbance frequency per se and time since last disturbance, because more frequently disturbed systems have also usually been disturbed more recently. Our understanding of the effects of repeated disturbances is therefore confounded by differences in successional processes.
3. We used in‐situ stream mesocosms to isolate and examine the effect of disturbance frequency on community composition. We applied substrate moving disturbances at five frequencies, with the last disturbance occurring on the same day across all treatments. Communities were then sampled after a recovery period of 9 days.
4. Macroinvertebrate community composition reflected the gradient of disturbance frequency driven by differential vulnerability of taxa to disturbance. Diversity metrics, including family‐level richness, decreased, reflecting a likely loss of functional diversity with increasing disturbance frequency. In contrast, overall abundance was unaffected by disturbance frequency as rapid recovery of the dominant taxon compensated for strong negative responses of disturbance‐vulnerable taxa.
5. We show that cumulative effects of repeated disturbances—not just the time communities have had to recover before sampling—alter communities, especially by disproportionately affecting rare taxa. Thus, the timing of past disturbances can have knock‐on effects that determine how a system will respond to further change.

## 1 INTRODUCTION

Disturbances can have strong effects on multiple levels of the community, either by altering whole‐community dynamics (e.g., depressing biomass) or through disproportional impacts on vulnerable taxa (Supp & Ernest, 2014). Frequency of disturbance is a key aspect of a community's disturbance regime, and can be conceptualized as two separate but related effects: the cumulative effects of repeated disturbances, and different time since last disturbance that generates varied recovery states. Disturbances exclude taxa from the community if they are poorly adapted to associated stressors (Cadotte & Tucker, 2017; Lebrija‐Trejos, Pérez‐García, Meave, Bongers, & Poorter, 2010); for example, taxa can be excluded directly by abiotic factors like temperatures outside a physiological tolerance, or indirectly if predation pressure is too high for a prey species to persist. As anthropogenic climate and land‐use change continue, we may see shifting community composition with increasing or decreasing frequency of disturbance events. Due to variation in tolerance to disturbances, we would expect that different thresholds for disturbance resistance lead to progressively more loss of individuals and taxa as disturbance frequency increases.

**Figure 1:**
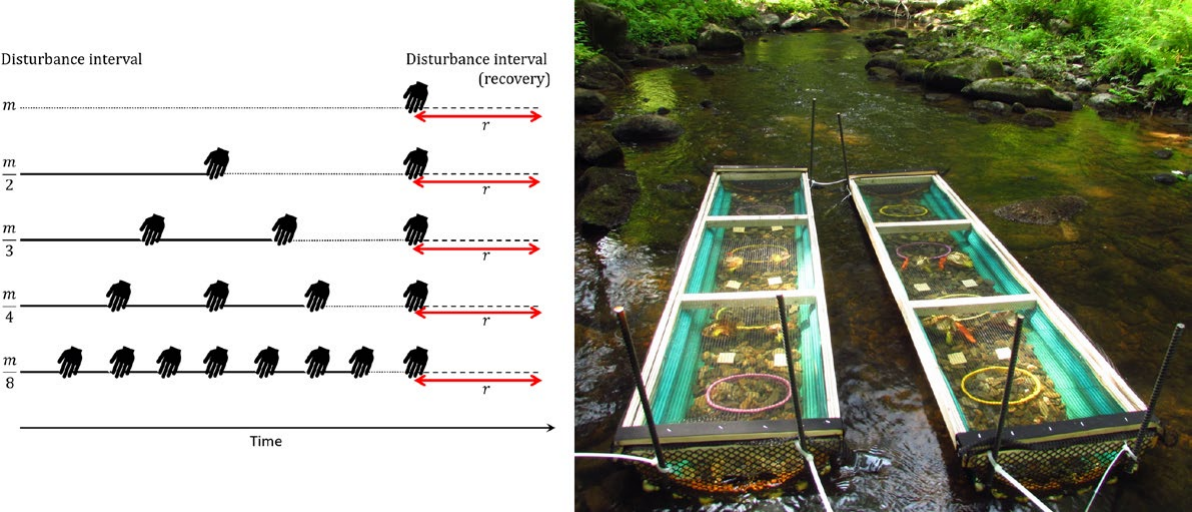
Experimental design and schematic of treatments. Stream mesocosms (right) contained gravel, sampling baskets, and leaf bags. They were manually disturbed (hand symbol) either 1, 2, 3, 4, or 8 times in the 29‐day initial manipulation period (*m*, August 2014) then left to recover for 9 days (*r*) to equalize time since last disturbance across treatments

In natural systems, alongside the cumulative effects of repeated disturbances we must consider the influence of time since last disturbance—or successional state—on communities, because community composition changes as communities recover (Clements, 1916; Gleason, 1917). More frequently, disturbed systems have, at any point in time, also usually been disturbed more recently, and thus are at different stages of recovery when sampled (Death & Winterbourn, 1995). These communities therefore reflect the sum of differential vulnerability of taxa to filtering events, and taxon‐specific colonization processes operating in the time since last disturbance. Therefore, empirical studies should isolate the role of repeated disturbances from time since last disturbance (Figure 1) to understand the causes of compositional changes.

Changing disturbance frequencies are a global concern. For example, fire suppression and river impoundments reduce disturbance frequency, and climate change is causing more frequent flooding and drought (Huntington, 2006). Consequently, many communities are being subjected to disturbance regimes outside of their historical norms. In streams, hydrological disturbances are among the most important drivers of community composition (Death & Zimmermann, 2005; Stanley, Powers, & Lottig, 2010). Flooding and resulting streambed movement impact organisms directly by inducing dislodgment and mortality (Holomuzki & Biggs, 2000; Lake, 2000), and indirectly through the removal of basal food resources (Zimmermann & Death, 2002) and by influencing the strength of competition and predation (McAuliffe, 1984). Although many stream organisms display behavioral, morphological, or life history adaptations that can help them persist through or avoid disturbances (Lytle & Poff, 2004), taxa differ in their ability to tolerate flood disturbances. Moreover, the traits that confer tolerance to disturbances are often reliant on life‐history transitions that are synchronized to either seasonally predictable disturbance regimes or environmental cues prior to the peak of disturbance effects (Lytle, Bogan, & Finn, 2008). Increasing frequency and intensity of hydrological extremes with climate change (Huntington, 2006; Palmer & Räisänen, 2002) may not be in accordance with the environmental conditions under which disturbance‐adapted stream taxa have evolved (Boersma, Bogan, Henrichs, & Lytle, 2013; Lytle & Poff, 2004) and are likely to have important community and ecosystem‐level consequences, such as intensification of predation rates when habitat size is reduced in refugia (Woodward et al., 2016). Thus, increases in the frequency of disturbance events are likely to result in declines in the abundance and persistence of disturbance‐intolerant taxa, along with associated changes in community richness and composition.

We focused on the effects of repeated disturbances using in‐situ mesocosms subjected to simulated flood disturbances, allowing precise control of disturbance frequency and time since last disturbance. These mesocosms (“channels”) were colonized from the surrounding stream environment. We then applied disturbances to these systems at varying frequencies, with the last disturbance occurring on the same date (after Peterson & Stevenson, 1992, Lake, Doeg, & Marchant, 1989). After a recovery period, we then quantified family‐level richness and community composition, as well as responses of individual families. Having the last disturbance occur on the same date enabled us to address the influence of disturbance frequency on community composition without the confounding influence of recovery status. We hypothesize that increasing disturbance frequency will lead to a corresponding loss of individuals and taxa, as progressively more disturbance‐intolerant taxa are lost in the system. If this hypothesis is supported, it will indicate that we need to consider historical disturbance regimes when predicting responses to future disturbances.

## 2 METHODS

The experiment was conducted in Pollard Brook (Edinburg, Maine, 45°10′28.5″ N, 68°38′13.6″ W), a small second‐order stream that drains a catchment dominated by wetlands and mixed conifer and broadleaf forest. The high volume of fine particulate organic matter (FPOM) and seasonal inputs of coarse detritus likely constitutes the main basal resource for the food web of this heavily shaded stream.

The stream mesocosms consisted of a 1.8 m long U‐shaped channel (Figure 1) constructed from PVC roofing sheets bent around a semi‐circular wooden frame. We capped the channels with ~20 mm mesh on the up‐ and downstream end and affixed them with a hinged shadecloth lid on top. Up‐ and downstream mesh had openings large enough to allow passage of most animals except large fishes (e.g., alewife and salmonids); small fish (black‐nosed dace) and crayfish (F: Cambaridae) over 5 cm long were observed within the channels. Fifteen channels were secured with steel rebar to the streambed on August 1, 2014, either placed singly or side‐by‐side in pairs with a ~1 m gap between them. Distances between channels and their up or downstream counterparts ranged from 5.3 to 30 m. Habitat structure was added in the form of 15 L of gravel (~3.5 cm diameter particles), four gravel‐filled plastic baskets arranged longitudinally, and four 10 g bags of maple leaf detritus to each channel on the day of installation.

We assigned five disturbance frequency treatments, with three replicates each, to channels over three randomized blocks reflecting upstream to downstream position in the 250 m stream reach. Over a 1‐month period (*m* in Figure 1), channels were disturbed either 0, 1, 2, 3, or 7 times by manually churning the gravel in a systematic pattern from upstream to downstream, followed by additional movements to ensure gravel was evenly spread through the channel. This procedure simulated the bed‐moving aspect of a flood disturbance (after Lake et al. (1989), McCabe and Gotelli (2000)). These disturbances can be categorized as “pulse” disturbances and are discrete events of abiotic stress in time (Lake, 2000). Nets with 500 µm mesh were placed downstream of each channel during each disturbance to collect dislodged individuals, which were preserved in 70% ethyl alcohol. This also precluded displaced individuals from recolonizing channels downstream. Every 3–4 days, we cleared leafy debris from the upstream end of all channels to maintain flow.

At the end of this 1‐month period (August 29, 2014), we removed two baskets from each channel, rinsed their contents over a 500 µm sieve, and preserved samples in 70% ethyl alcohol. Cleaned baskets were returned to their channel to maintain a homogeneous environment through time. All channels were then disturbed once using the manual disturbance procedure described above to standardize the recovery period among treatments (*r* in Figure 1). This design enabled us to compare the effect of disturbance frequency on channel communities with the same time since last disturbance. The experiment ended after a 9‐day recovery period, upon which the two previously unsampled baskets were removed to subsample communities and detritus.

### 2.1 Laboratory analysis

The majority of individuals were identified under 10–60× magnification to family level using Merritt, Cummins, and Berg (2008) as immature stages and small body sizes prevented consistency in further identification. Acari and Gastropoda were identified to subclass and class, respectively. Oligochaetes were too damaged and fragmented by the disturbances to count accurately, so were excluded from the dataset. Fragments indicated that oligochaetes were broadly distributed across treatments but occurred in low abundance, so it is unlikely their exclusion from our dataset would have altered the outcome of our analyses.

After separating invertebrates from the sample, we used a series of nested sieves to retain fine particulate organic matter (FPOM, 63 µm – 1,000 µm). FPOM was oven‐dried (60°C, >72 hr), weighed, ashed in a muffle furnace for 2 hr at 550°C, and then reweighed to determine ash‐free dry mass.

### 2.2 Data analysis

Two baskets were sampled from each channel on 29th August and 7th September. The first sample (29th August) was only analyzed for number of chironomids and total FPOM, not family‐level community composition, due to time constraints. The community data from both baskets were pooled, as was the total FPOM. All abundances and FPOM ash‐free dry mass data therefore reflect the contents of two baskets, and not the entire stream channel.

We analyzed the effects of disturbance frequency on taxon richness, evenness, and total abundance using linear mixed effects models (package nlme in R). We regressed the response variables against the number of disturbances a channel experienced over the duration of the experiment (August 1, 2014, – September 7, 2014) and used experimental block as a random term. We used the R package piecewiseSEM (Lefcheck, 2016) to calculate *R* ^2^ values from mixed effects models (following Nakagawa & Schielzeth, 2013). In practice, because undisturbed channels were subject to the standardized disturbance that began the period, the frequency of disturbance varied from 1 to 8 among the five treatments. Response variables were transformed in a manner appropriate to meet assumptions of normality, and a significance threshold of α = 0.05 was employed.

Rarefied familial richness was calculated with the function “rarefy” in R package vegan using the minimum per‐channel abundance across all channels (Oksanen et al., 2016) —73 individuals. Pielou's equitability was calculated as a metric of taxonomic evenness by dividing Shannon index by ln (# of families in a sample).

We assessed changes in community composition with partial redundancy analysis (pRDA) on a Hellinger‐transformed macroinvertebrate abundance matrix. Our initial RDA included both disturbance frequency (5‐level factor) and experimental block as constraining factors. Permutation tests (999 iterations) of the reduced model indicated significant effects of both block (*F*_2,8_ = 2.85, *p* < 0.002) and disturbance frequency (*F*_4,8_ = 2.54, *p* < 0.003) on community composition. Subsequent partial RDA focused on the effect of disturbance frequency by including block as a conditioning factor. Again, significance was tested with permutation tests with 999 permutations of the reduced model.

## 3 RESULTS

**Figure 2:**
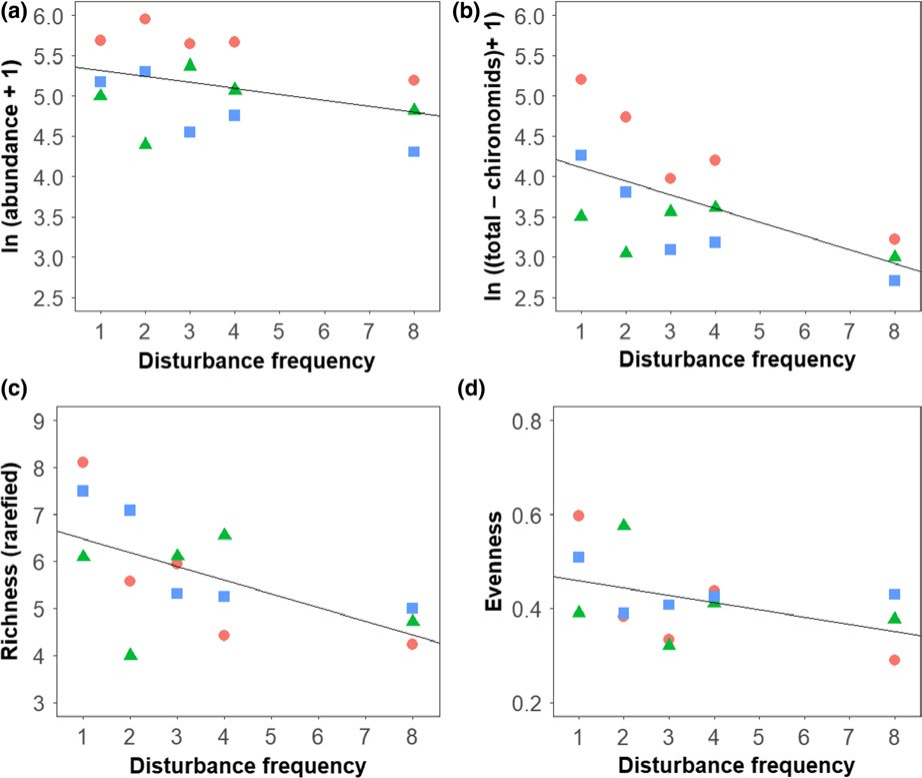
The influence of the frequency of streambed‐moving disturbance on diversity. (a) Total abundance of invertebrates, slope = −0.07, *p* = 0.048; (b) abundance of all invertebrates barring the dominant taxon (chironomids) within in‐situ stream channels, slope = −0.18, *p* = 0.002; (c) rarefied richness, slope = −0.29, *p* = 0.017; (d) Pielou's equitability, slope = −0.02, *p* = 0.098. Symbols and colors indicate experimental block: upstream (red circle), mid‐stream (green triangle) and downstream (blue square)

Disturbance responses varied by the taxon studied, with the most pronounced responses in rare taxa and no detectable response within the overwhelmingly dominant taxon. Total abundance did vary significantly with disturbance frequency, but the effect size was small (slope = −0.07, *p* = 0.048, *R* ^2^ = 0.69; Figure 2a). However, when the most dominant taxon, chironomid midges (73% of abundance) were removed from analyses, we observed a stronger decline in the abundance of non‐chironomid taxa (mites, snails, and the remaining 26 insect families) with disturbance frequency (slope = −0.18, *p* = 0.002, *R* ^2^ = 0.74; Figure 2b).

**Figure 3:**
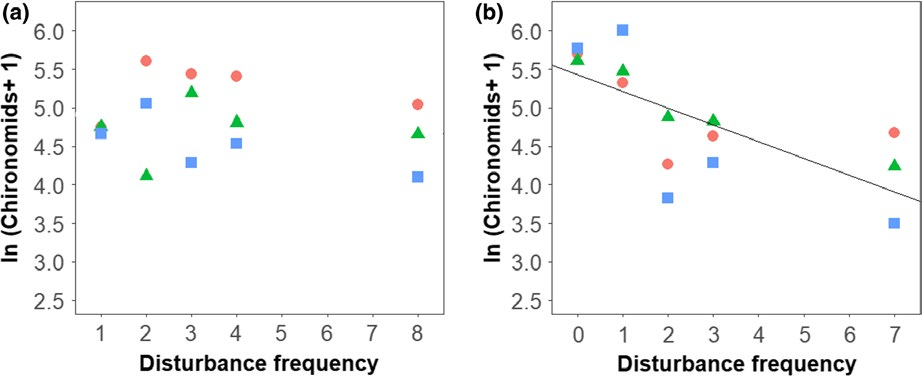
Abundance of the dominant taxon, chironomid midges. (a) September 7, 2014, *p* = 0.436, and (b) August 29, 2014, immediately before final disturbance, slope = −0.217, *p* = 0.004. Symbols as in Figure 2

The dominant taxon, Chironomid midges, showed no abundance trend with disturbance frequency (*p = *0.436), though their abundance before the final disturbance—that is, when treatments also differed in time since the last disturbance—showed a negative relationship with disturbance frequency (slope = −0.22, *p = *0.004, *R* ^2^ = 0.49; Figure 3), indicating that time since last disturbance was the main determinant of the abundance of the dominant taxon. In contrast, Heptageniidae (flat‐headed mayflies) were the second most abundant taxon and were strongly negatively affected by increasing disturbance frequency (slope = −0.26, *p = *0.004, *R* ^2^ = 0.66), whereas neither swimming mayflies (Baetidae) nor Calopterygidae damselflies were affected by disturbance frequency (*p* = 0.32 and *p = *0.80, respectively). The remaining taxa (Table S1) that encompassed only 4% of individuals were also strongly affected by disturbance (slope = −0.21 *p* = 0.005, *R* ^2^ = 0.63). Twelve of the 29 invertebrate taxa observed were singletons.

**Figure 4:**
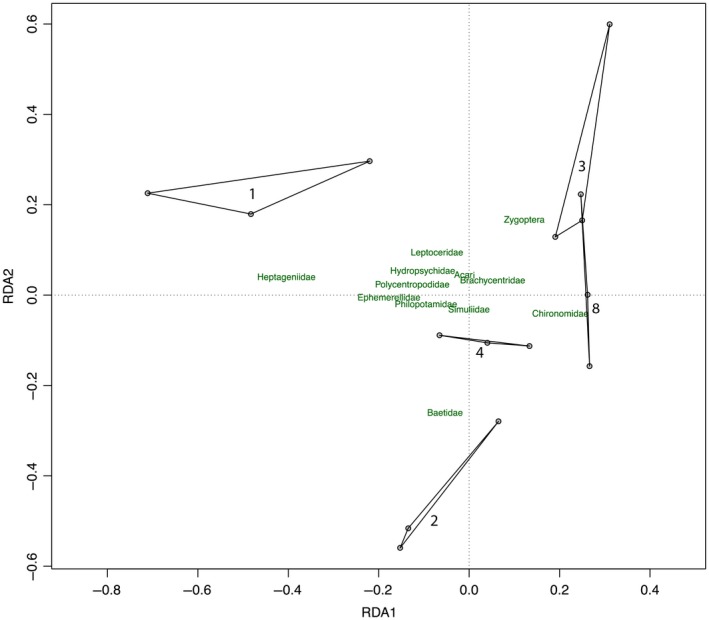
Partial redundancy analysis of Hellinger‐transformed macroinvertebrate abundance matrix. The pRDA was conditioned on a factor that accounted for the stratification of treatments across three spatial blocks. Polygons encompass the three replicates of each for disturbance frequency treatment, with labels indicting the total number of disturbances. A subset of 12 of 29 taxa with the strongest axis loadings are shown for clarity

Partial redundancy analysis provided further support for significant compositional changes under increased disturbance frequency (Figure 4; Permutation ANOVA, *F*_4,8_ = 2.54, *p* = 0.005). Channels subject to only one disturbance were characterized by heptageniid mayflies, several trichopteran families, and numerous other taxa, whereas communities subject to high disturbance frequencies consisted mostly of chironomids (Figure 4). Baetidae and Zygoptera were abundant in moderately disturbed treatments (Figure 4).

Rarefied taxonomic richness declined significantly as disturbance frequency increased (slope = −0.29, *p = *0.020, *R* ^2^ = 0.35; Figure 2c). Disturbance frequency had a marginally non‐significant effect on taxonomic evenness (Figure 2d), with a general trend of declining evenness with greater disturbance frequency.

**Figure 5:**
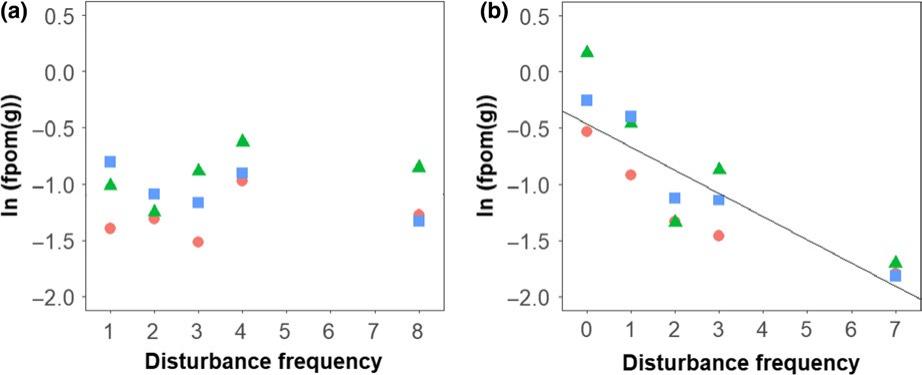
Ash‐free dry mass of fine particulate organic matter (FPOM) in stream channels that were disturbed at different frequencies. Data presented are from (a) September 7, 2014 in which all channels have equal time since last disturbance; *p* = 0.960, and (b) August 29, 2014, prior to the last disturbance; slope = −0.21, *p* < 0.001. Symbols as in Figure 2

Fine particulate organic matter (the basal resource) was strongly affected by disturbance frequency on 29th August, prior to the final disturbance (slope = −0.21, *p < *0.001, *R* ^2^ = 0.76; Figure 5b). However, this pattern was no longer apparent on 7th September following the standardized disturbance that reset communities to begin the 9‐day recovery period (*p = *0.960; Figure 5a), indicating that time since last disturbance affects FPOM accumulation, but disturbance frequency per se does not.

## 4 DISCUSSION

Our experiment shows that even when communities were last disturbed at the same point in time, their frequency of past disturbances leaves a legacy on community composition by disproportionately affecting rare taxa. Several studies have quantified the cumulative effect of disturbance frequency and time since last disturbance on communities (e.g., Thomson, 2002, Joubert, Pryke, Samways, Stewart, & Dennis, 2016, Death, 1996, McCabe & Gotelli, 2000). Our novel approach of separating these mechanisms demonstrates that more frequent disturbances can alter community composition not only by interrupting and resetting colonization but also by changing the intrinsic habitat suitability through the direct and indirect effects of disturbance events.

### 4.1 Influence of disturbance frequency on abundance of taxa

One of the main community‐level effects of disturbances is a reduction in overall abundance of individuals (McMullen & Lytle, 2012; Supp & Ernest, 2014), whether through direct displacement of individuals, or indirectly reduction in resources or shifts in species interactions such as competition and predation. A natural extension is that a series of disturbances might reduce abundance more than a single disturbance event. This hypothesis was supported by a slight, but significant, reduction in overall abundance with increasing disturbance frequency at the community level (Figure 2a). However, responses to disturbances at the taxon‐level are generally more pronounced than at the community level (Supp & Ernest, 2014), as was the case in our experiment. While the dominant taxon, chironomids (comprising 70% of overall abundance) did not decline with disturbance frequency, the abundance of the remaining taxa (mites, snails, and the remaining 26 insect families) decreased strongly with disturbance (Figure 2b) and we observed significant variation in community composition across the gradient of disturbance frequency (Figure 4). This suggests that vulnerability to repeated disturbances varies among taxa and was evident across the broader range of species present in this community. Moreover, the response of dominant taxa may mask the magnitude of the complex, taxon‐specific responses occurring in the remainder of the community if we fail to look below the community scale.

Taxon‐scale traits explain the differences between disturbance responses in the dominant taxon and the rarer taxa. For a taxon to be buffered against disturbance, taxa can be either resistant, in that they are unaffected by the stressors that occur during the disturbance event, or resilient, in that their populations recover quickly (Pimm, 1984). Data collected immediately prior to the final disturbance, when time since last disturbance also varied, show a sharp decline in chironomid density with increasing disturbance frequency whereas data collected 9 days later at the final sampling date show no trend (Figure 3). This indicates that chironomid densities are more influenced by colonization in the time since last disturbance than resistance to the cumulative impacts of repeated disturbances. Numerically dominant species tend to have smaller body sizes (Cohen, Jonsson, & Carpenter, 2003), as in chironomids here—which can also be associated with fast recolonization, growth, and reproduction (Pianka, 1970), traits which could link dominance to high resilience to perturbation. Similar trends have been observed in disturbance experiments in alpine streams (Maier, 2001), Afromontane grasslands (Joubert et al., 2016), and brackish wetlands (Kettenring, Whigham, Hazelton, Gallagher, & Weiner, 2015), suggesting the overriding influence of colonization rate on disturbance responses may be a general phenomenon. In addition, the same pattern was observed in detritus in this experiment (Figure 5), supporting the similar, passive dispersal mechanism for both fine particulate organic matter and chironomid midges.

We also observed evidence for the importance of resistance to multiple disturbance events in the less common taxa. The second most abundant taxon (heptageniid mayflies) responded negatively to more frequent disturbance, and this pattern remained detectable even after the 9‐day recovery period. These results are suggestive that this taxon had some resistance to single disturbances—enough to allow some individuals to survive—but they were vulnerable to repeated substrate disturbance. Studies of heptageniids in natural spates have shown that individuals <2 mm were among the taxa most affected by a single flood, but also that their abundances recovered to pre‐flood values within 8 days because smaller, early‐instar individuals replaced the previous residents (Maier, 2001). If smaller individuals are less resistant to disturbance, and are replaced by even smaller, earlier instar individuals, the population may become more vulnerable with successive disturbances. This is a potential mechanism for the negative disturbance frequency‐abundance relationship seen in many taxa here, especially because many individuals sampled in this experiment were less than 2 mm in length, and the early instars of many taxa display higher dispersal rates than their older and larger conspecifics (Hieber, Robinson, & Uehlinger, 2003). Future studies including body size might elucidate the drivers of disturbance responses.

Like heptageniid mayflies, rare taxa (~4% of total abundance), also declined with disturbance frequency. This suggests that many of these taxa were able to survive a single disturbance, but that each successive disturbance further reduced population sizes and increased the chance of local extirpation. In contrast, baetid mayflies, which are strong swimmers (Peckarsky, 1996), were unaffected by disturbance; this may be due to better refugium‐seeking strategies and higher mobility (Maier, 2001). This pronounced taxon‐specific variation in responses and the overriding role of dominant taxon highlights that both colonization/resilience and resistance are likely mechanisms for how disturbance mediates community composition and diversity.

### 4.2 The relationship between diversity and disturbance frequency

The differential susceptibility of taxon abundance to disturbance was reflected in the decline in rarefied family‐level richness in more frequently disturbed communities (Figure 2c). The time to local extirpation depends on the magnitude of disturbance effects, how frequently disturbances occur, and whether a population's growth rate can replenish its numbers fast enough between disturbances to overcome these two negative influences (Lande, 1993). Thus, less abundant taxa in a community with lower population growth rates will be more liable to local extirpation under recurring disturbances (Cleland et al., 2013). This stochastic effect of disturbance can therefore reduce richness, regardless of variation in disturbance susceptibility traits among taxa.

There is also strong experimental evidence that deterministic processes linked to species' traits, such as growth rate, produce differential susceptibility to frequency and intensity of disturbances (Haddad et al., 2008). This is supported by the theoretical negative relationships between population growth rate and disturbance‐induced extinction (Lande, 1993). The slight decline in whole‐community abundance coupled with greater losses in some of the rarer taxa led to removal of whole families from the mesocosm communities. Factors like body size and mobility might help to explain which taxa were most susceptible (Maier, 2001). Data with higher temporal resolution—for example, tracking recovery trajectories over time (Lake et al., 1989)—as well as specific data on taxonomic traits and individual body size may help future studies determine the relative importance of stochastic taxon loss or deterministic, fitness‐based changes in community composition.

To our knowledge, this is the first manipulative experiment that has shown a significant negative effect of disturbance frequency per se on taxonomic richness when the confounding influence of time since last disturbance has been removed. Here, we show that these responses were observed at the family‐level, and we do not contend that these data serve as a proxy for species richness. Nevertheless, compared with loss of species, loss of entire families is more likely to reflect a reduction in functional richness alongside taxonomic losses, because traits are generally highly conserved within aquatic invertebrate families. (Poff et al., 2006). For example, functional feeding groups for aquatic macroinvertebrates are generally shared within a family (Merritt et al., 2008), so while at the species level it is possible that functional redundancy might mediate some of the effects of lower richness, this is less likely at the family level.

These responses may shed some light on the mechanisms behind studies of disturbance frequency which did not standardize for time since last disturbance. For example, McCabe and Gotelli (2000) found that rarefied richness actually increased with greater disturbance frequency, while the absolute number of taxa decreased, reflecting changes in abundance over successional time. Therefore, our novel result expands on previous findings (Lake et al., 1989; McCabe & Gotelli, 2000) and has explicitly shown that disturbance frequency, not time since last disturbance, is responsible for our observed declines in richness. More broadly, we suggest that partitioning out these two drivers of diversity‐disturbance frequency relationships may hold the key to understanding variation in responses across systems that have been subjected to different historical disturbance regimes.

### 4.3 Disturbance frequency as a mechanism structuring communities

Though it is apparent that richness and the abundance of many taxa decreased with disturbance frequency, we did not explicitly test the mechanisms behind this decline. Disturbance can act on invertebrates in a patch either directly by inducing downstream drift or causing mortality (Matthaei, Uehlinger, & Frutiger, 1997), or indirectly by affecting resources (Death & Zimmermann, 2005) that can also alter patterns of competitive exclusion (McAuliffe, 1984). Our study stream is heavily shaded in the summer and contains high amounts of fine particulate organic matter which was entrained by gravel within days of installation of fresh channels. However, as in natural floods, disturbance of the substratum dislodged FPOM out of the mesocosms, which is reflected in the difference in FPOM ash‐free dry mass before and after disturbance (Figure 5). While there was no apparent legacy effect of historical disturbance frequency on end‐date FPOM, indirect effects mediated by the loss of this resource may have played a role in community composition before the final disturbance occurred. However, several observations suggest this mechanism was unlikely. Firstly, chironomids that primarily feed on fine detritus (Romito, Eggert, Diez, & Wallace, 2010) showed no legacy of disturbance frequency. In contrast, heptageniids, which primarily feed on algal biofilms rather than FPOM (Cummins & Klug, 1979), were strongly affected by disturbance, suggesting a direct effect of bed‐moving on mortality. However, separating direct and indirect effects of disturbance is challenging (Death, 2003) and further investigation is needed to determine whether a legacy of resource changes with disturbance frequency is an important aspect of stream community structure and composition.

## 5 CONCLUSIONS

This study provides direct evidence that disturbance frequency affects community composition through cumulative stresses associated with repeated disturbances. Although numerous experiments and surveys that utilize natural gradients of disturbance have shown repeated disturbances can influence community composition, they cannot unravel the relative influence of disturbance frequency and time since last disturbance. By controlling for time since last disturbance, our experiment revealed that disturbance frequency itself can affect diversity—even detectable on coarse taxonomic scales. Teasing apart the relative importance of disturbance frequency itself versus the successional processes that operate in the time since the last disturbance will clarify the mechanisms underlying disturbance responses in general, and help understand ecosystem responses to shifting disturbance regimes as climate and land‐use change.

## CONFLICT OF INTEREST

None declared.

## AUTHOR CONTRIBUTIONS

JH and HG designed the experiment and supervised data collection. All authors conducted field data collection and labwork, including invertebrate identification. JH, HG, and JM collated, analyzed and presented the data.

## Data Availability

The data associated with this manuscript are archived in Dryad Digital Repository https://doi.org/10.5061/dryad.bv1d63h.
