# Supplementary material for: Repeat disturbances have cumulative impacts on stream communities
Source: Ecol Evol. 2019 Feb 14;9(5):2898–906. doi: 10.1002/ece3.4968 (PMC6405533; doi:10.1002/ece3.4968)
Supplement: Supplementary file 1 [file ECE3-9-2898-s001.docx]

# **APPENDIX: SUPPLEMENTAL DATA**

Table S1 List of total abundance of taxa found in stream mesocosms. Data are summed across all subsamples collected on 7^th^ September 2014. *indicates an unidentified beetle larva that was likely a unique family.

| **Taxon** | **Abundance** | **% Grand total abundance** |
| --- | --- | --- |
| Chironomidae | 2056 | 73.30 |
| Heptageniidae | 291 | 10.37 |
| Baetidae | 215 | 7.66 |
| Zygoptera | 94 | 3.35 |
| Hydropsychidae | 37 | 1.32 |
| Leptophlebiidae | 22 | 0.78 |
| Ephemerellidae | 16 | 0.57 |
| Philopotamidae | 16 | 0.57 |
| Polycentropodidae | 8 | 0.29 |
| Mollusca | 8 | 0.29 |
| Leptoceridae | 6 | 0.21 |
| Aeshnidae | 5 | 0.18 |
| Simuliidae | 4 | 0.14 |
| Perlodidae | 4 | 0.14 |
| Brachycentridae | 2 | 0.07 |
| Taeniopterygidae | 2 | 0.07 |
| Mite | 2 | 0.07 |
| Isonychiidae | 1 | 0.04 |
| Tricorythidae | 1 | 0.04 |
| Caenidae | 1 | 0.04 |
| Molannidae | 1 | 0.04 |
| Hydroptilidae | 1 | 0.04 |
| Corixidae | 1 | 0.04 |
| Tipuliidae | 1 | 0.04 |
| Coleoptera Larva* | 1 | 0.04 |
| Dytiscidae Larva | 1 | 0.04 |
| Leuctridae | 1 | 0.04 |
| Perlidae | 1 | 0.04 |
| Chloroperlidae | 1 | 0.04 |
| **Total abundance** | **2805** | **100%** |
